# Effects of recreational football on body composition, cardiometabolic health, and functional performance in children and adolescents: a systematic review and meta-analysis

**DOI:** 10.3389/fphys.2025.1707395

**Published:** 2026-01-16

**Authors:** Yanzhao Lei, Yanmei Ding, Bo Wang, Hengzhi Deng, Mingyue Yin, Kai Xu, Hengxian Liu, Meiling Tao, Yanfeng Li, Yilin Zhang, Yuhang Liu, Fanhao Meng, Hansen Li, Xing Zhang, Bitai Wu

**Affiliations:** 1 Shanghai Sipo Polytechnic, Shanghai, China; 2 Shanghai Civil Aviation College, Shanghai, China; 3 Jilin University, Changchun, China; 4 University of Malaya, Kuala Lumpur, Malaysia; 5 Shanghai University of Sport, Shanghai, China; 6 Edith Cowan University, Joondalup, WA, Australia; 7 Capital University of Physical Education and Sports, Beijing, China; 8 Tianjin University of Sport, Tianjin, China; 9 Beijing Sport University, Beijing, China; 10 Sichuan Agricultural University, Ya’an, China; 11 University of Granada, Granada, Spain; 12 Changsha University of Science and Technology, Changsha, China

**Keywords:** adolescents, cardiometabolic health, children, functional performance, recreational football

## Abstract

**Purpose:**

The systematic review and meta-analysis aimed to evaluate the effects of recreational football on body composition, cardiometabolic health, and functional performance in children and adolescents. Additionally, it explored potential moderators through subgroup analyses.

**Methods:**

A systematic search was conducted in six databases in May 2025. A random-effects model was employed for the meta-analysis, and effect sizes were reported as standardized mean differences (SMD, Hedges’ *g*). Subgroup and sensitivity analyses were conducted to examine sources of heterogeneity.

**Results:**

A total of 20 studies (N = 2,906; age range: 8–17 years) were included. Of the participants, 1,524 (52.44%) were male, 1,174 (40.40%) were female, and 208 (7.16%) did not clearly report their gender. Recreational football significantly reduced BMI (SMD = −0.13 [−0.24, −0.02]), body fat percentage (SMD = −0.37 [−0.63, −0.11]), and waist circumference (SMD = −1.38 [−2.65, −0.11]), with a slight increase in lean mass (SMD = 0.13 [0.02, 0.24]). It also reduced mean arterial pressure (SMD = −1.06 [−2.03, −0.10]), systolic blood pressure (SMD = −0.71 [−1.19, −0.23]), and triglycerides (SMD = −0.95 [−1.74, −0.15]), while having no effect on diastolic blood pressure, resting heart rate, VO_2_peak, blood glucose, or cholesterol. Additionally, it improved interval endurance (SMD = 0.15 [0.04, 0.25]), sprint speed (SMD = −0.72 [−1.22, −0.22]), standing long jump (SMD = 0.53 [0.10, 0.97]), and balance (SMD = 0.84 [0.21, 1.46]), but had no effect on vertical jump. Subgroup analyses showed greater reductions in BMI (g = −0.54) and body weight (g = −0.89) in overweight/obese individuals, and significant weight improvement in adolescents >12 years (g = −1.35). Longer interventions (≥12 weeks) and higher frequencies (>2 sessions/week) were associated with greater body fat reduction (g = −0.82 and g = −0.74), with reductions in resting heart rate observed mainly in interventions ≥12 weeks (g = −0.72). According to the GRADE assessment, the overall quality of evidence was rated as low to very low.

**Conclusion:**

Recreational football is efficacious in improving body composition, select cardiometabolic risk factors, and physical performance in children and adolescents, especially individuals classified as overweight or obese. Even with limitations in sample size, intervention diversity, and methodological quality, resulting in an overall low to low quality assessment of the evidence, the comprehensive evidence still provides preliminary quantitative support for incorporating recreational football into youth health promotion; future efforts will require larger samples, standardized protocols, and rigorous design to enhance the strength of the evidence. Based on existing evidence, a reference protocol may consist of a 12-week program with 2–3 weekly sessions (45–60 min each), including a FIFA 11+ warm-up, 3–4 bouts of 4–6 min at up to 80% HR_max_ with 2-min recovery intervals, and a cool-down.

**Systematic Review Registration:**

https://www.crd.york.ac.uk/PROSPERO/view/CRD420251106734, identifier CRD420251106734.

## 1 Introduction

Recently, global physical activity levels among children and adolescents have steadily declined, while overweight and obesity have emerged as increasingly serious public health concerns (Guthold et al., 2020). Global surveillance indicates that approximately 80% of adolescents fail to meet the recommended ≥60 min per day of moderate-to-vigorous physical activity (MVPA), and in 2020, an estimated 340 million children and adolescents (5–19 years) had overweight or obesity (The Global Status Report, 2022). Moreover, a growing body of evidence indicates that the co-occurrence of excess adiposity and inadequate MVPA among youth is associated with early metabolic abnormalities and an increased risk of developing cardiovascular disease over the lifespan (Kallio et al., 2021). Therefore, scalable and effective strategies to increase MVPA are a public health priority.

Among the various strategies to improve physical inactivity and metabolic health in children and adolescents, regular physical exercise is recognized as one of the most effective approaches (Swift et al., 2014). In recent years, recreational football has attracted growing attention due to its engaging nature and comprehensive training benefits (Krustrup et al., 2018). Compared to high-intensity interval training protocols that are typically based on repetitive single-mode exercises such as running or cycling, recreational football offers a more diverse range of physical activities and enhances social interaction and enjoyment in a team-based and competitive environment, effectively reducing dropout rates and increasing participant engagement (Zouhal et al., 2020). Unlike competitive football, which focuses on competitive results, recreational football has flexible rules and lower skill requirements, making it easier to participate and suitable for students of varying fitness and experience levels. Smaller fields and fewer players improve the efficiency of field and personnel utilization and facilitate promotion through group rotations or school and community programs (Sciences U of SDF of H, 2025). Moreover, its small-field format and frequent changes of direction engage large muscle groups across the body, strongly stimulating both aerobic and anaerobic systems and thereby enhancing endurance, explosive strength, and balance (Ahmad et al., 2025).

A growing body of systematic reviews has demonstrated that recreational football is highly effective in improving body composition and metabolic health. For example, one systematic review reported significant improvements in increases in VO_2_peak by 7%–16%, bone mineral density by 5%–13%, and reductions in systolic blood pressure (SBP) by 7.5%, diastolic blood pressure (DBP) by 10.3%, low-density lipoprotein cholesterol (LDL) by 15%, and body fat mass by 2%–5% (Zouhal et al., 2020). Furthermore, a meta-analysis of studies targeting overweight or obese individuals also showed that recreational football significantly improved body composition (SMD = −1.31 to −0.40) and cardiovascular metabolic parameters (SMD = −0.91 to 0.81), providing strong evidence for interventions in this special population (Li et al., 2025). Another meta-analysis found that, compared with maintaining usual activity, recreational football significantly reduced blood pressure (−3.9 to −4.2 mmHg), resting heart rate (−6 bpm), and fat mass (−1.7 kg), while improving countermovement jump (CMJ) (+2.6 cm) (Milanović et al., 2019). In untrained children and adolescents, one systematic review showed improvements in aerobic fitness and certain cardiovascular indicators, but no significant changes in body composition (Clemente et al., 2022). It is noteworthy that this review did not perform a meta-analysis to quantify the intervention effects and instead provided only a qualitative synthesis. Furthermore, a recent meta-analysis reported that recreational football improved VO_2_peak (SMD = 0.12), blood pressure (MD = −1.26 to −3.85 mmHg), and triglycerides (TG) (MD = −30.34 mg dL^-1^) in untrained children and adolescents, but showed no significant improvements in body composition or other cardiometabolic markers (Gómez-Álvarez et al., 2024). In summary, while previous reviews have suggested potential benefits, they have generally been limited by small included studies and sample sizes, non-standard reporting of intervention prescriptions and intensity, incomplete coverage of outcome indicators, and insufficient systematic evaluation of heterogeneity and bias.

Additionally, although existing studies have conducted subgroup analyses, these have primarily focused on participant characteristics and the duration of the intervention. Reporting of core intervention components, including training intensity, session length, and session frequency, is both insufficient and inconsistent, with no standardized definitions. Although previous studies have demonstrated that a frequency of 3–5 days per week is more effective for improving cardiorespiratory fitness and lowering blood pressure (Garber et al., 2011), this key variable, despite its central role in designing school-based physical education and health promotion programs, remains insufficiently investigated in the current literature. Overall, recreational football has demonstrated considerable potential for promoting cardiometabolic health and physical fitness in children and adolescents. However, existing studies suffer from small sample sizes, non-standardized intervention protocols, incomplete outcome measures, and inconsistent assessments, particularly regarding intervention frequency and functional performance (e.g., sprint speed, endurance, and jumping ability). These limitations hinder the development of precise, evidence-based exercise recommendations for youth in school or the community.

Therefore, this meta-analysis aims to synthesize the available evidence on the effects of recreational football on body composition, cardiometabolic health, and functional performance in children and adolescents, and to explore potential moderators, including population characteristics (e.g., weight status) and exercise prescription (e.g., frequency). We hypothesize that its effects on the three category outcomes are both statistically significant and modulated by population characteristics and dose-response factors. The findings are intended to provide evidence-based guidance for school physical education curricula and youth health promotion programs.

## 2 Methods

This meta-analysis was conducted and reported following the 2020 PRISMA (Preferred Reporting Items for Systematic Reviews and Meta-Analyses) guidelines (Page et al., 2021) (Supplementary Appendix SA), and the study was pre-registered in the PROSPERO database (registration ID: CRD420251106734) on 23 July 2025.

### 2.1 Information sources and search strategy

Database searches were conducted in PubMed, Web of Science, the Cochrane Library, and three Chinese databases: CNKI (China National Knowledge Infrastructure), the VIP Database for Chinese Technical Periodicals, and Wanfang Data. No restrictions were applied regarding the publication date, participant characteristics, or language, provided that the title and abstract were available in English or Chinese. To ensure comprehensiveness, supplementary searches were conducted by filtering the reference list, performing citation tracking, and using the “similar articles” function in the database. The searches were conducted on 1 May 2025, and terms were formulated using the PICOS framework and categorized into two main concepts: population (“adolescents”) and intervention (“recreational football”), along with related terms (Supplementary Appendix SC).

### 2.2 Selection process

Deduplication of the retrieved records was performed manually by independent reviewers (BW and MYY) via EndNote X9. The deduplicated records were then exported and provided to two independent researchers, who screened the titles and abstracts of all articles against predefined inclusion and exclusion criteria. In cases of disagreement, the two reviewers met to re-examine the eligibility criteria and resolve discrepancies through discussion. If a consensus could not be reached, a third independent researcher (YZL) was consulted to make the final decision on inclusion. The same pair of independent reviewers then conducted a full-text review to determine final eligibility. Cohen’s k score was used to assess interrater reliability, revealing an initial agreement of 0.82 between the 2 reviewers (BW and MYY), indicating almost agreement. In the event of disagreement at this stage, the same adjudication protocol was applied. Additional potentially relevant sources included the reference lists of previous systematic reviews in the field and articles identified through the research team’s domain expertise that met the inclusion criteria but were not captured by the initial database search.

### 2.3 Eligibility criteria

The inclusion and exclusion criteria were developed according to the Population, Intervention, Comparison, Outcome, Study design (PICOS) framework, as detailed below.

The inclusion criteria for the population (P) encompassed studies involving children and adolescents (aged <18 years) from the general population, regardless of health status. Studies focusing on individuals engaged in organized, competitive athletic training, such as youth football players or children and adolescents participating in systematic sports club training aimed at enhancing athletic performance, were excluded. Similarly, studies involving adult participants (aged ≥18 years) were also excluded.

Intervention (I): The intervention was recreational football training, defined as small-scale (e.g., 4v4), non-competitive team football activities conducted on smaller pitches. The primary objective was to promote health and improve physical fitness, and it did not involve leagues, qualifying matches, or knockout stages; rankings or results were not recorded; and there were no selections or prize money awarded. Training did not focus on pre-match preparation or improving competitive performance, and there was no systematic technical or tactical instruction or periodic training schedule. To ensure consistency, we included only studies with interventions lasting at least 2 weeks and occurring at least twice a week.

Comparison (C): Comparators included non-exercise control, waiting list control, placebo, or usual care, such as nutritional advice or general lifestyle recommendations without structured exercise. These comparators were considered appropriate because they represent minimal or non-physical activity-based interventions commonly used in both clinical and community settings, especially among youth.

Outcome (O): Included studies were required to report at least one health-related outcome, including but not limited to: body composition indicators (e.g., weight, body fat percentage); cardiovascular health (e.g., blood pressure, resting heart rate, blood glucose, insulin sensitivity, lipid profile); functional performance (e.g., CMJ, balanced performance).

Study Design (S): Eligible studies were longitudinal controlled intervention trials, including randomized controlled trials (RCTs) and nonrandomized controlled trials (non-RCTs), with quantitative pre- and postintervention assessments, and reported between-group comparisons.

Studies were excluded if they lacked a control group (i.e., single-arm designs), failed to report data separately for children or adolescents, or involved interventions unrelated to recreational football, such as other exercise modalities, psychological interventions, or standalone dietary programs. Additional exclusion criteria included nonoriginal research formats (e.g., reviews, methodological articles, conference abstracts, gray literature, case reports, or qualitative studies), studies lacking postintervention outcome data or with insufficient information for effect size extraction, and duplicate publications or datasets, in which case only the version with the most complete and relevant data was retained.

### 2.4 Data extraction

Data extraction was carried out by the same two reviewers (BW and MYY) who conducted the screening process, via a customized extraction worksheet developed in Microsoft Excel, prior to the full-text review phase. Both reviewers independently extracted the following information from each included study: author and publication details, study design and characteristics, participant demographics, intervention protocols, and outcome assessments. A third reviewer (YZL) performed an additional round of cross-checking to verify the accuracy of the data. In cases of disagreement, a fourth independent researcher was consulted to reach a consensus through discussion. If data were missing or presented only in graphical form, the corresponding authors were contacted to request the required information. When the author’s contact was unsuccessful and the data remained available only in figure format, quantitative values were extracted via WebPlotDigitizer version 4.1. If the missing data could not be retrieved through either author contact or graphical extraction, the study was excluded from the final quantitative analysis.

### 2.5 Risk of bias and quality of methods assessment

The risk of bias was assessed independently by two reviewers via the Cochrane Collaboration’s risk of bias tool 2 (RoB 2). This tool evaluates bias across multiple domains, including random sequence generation, allocation concealment, the blinding of participants and personnel, the blinding of the outcome assessment, incomplete outcome data, selective outcome reporting, and other sources of bias. Disagreements between reviewers were resolved through discussion whenever possible. If a consensus could not be reached, a third independent reviewer was consulted for adjudication. For nonrandomized studies, the risk of bias was evaluated via the Risk of Bias in Nonrandomized Studies of Interventions (ROBINS-I) tool, which assesses seven domains of bias: confounding, the selection of participants, the classification of interventions, deviations from the intended interventions, missing data, measurement of outcomes, and the selection of the reported result. Additionally, the Physiotherapy Evidence Database (PEDro) scale was used to assess the methodological quality of the included studies. The PEDro scale rates studies on a scale from 0 to 10, with scores of ≥6 indicating high quality, scores of 4–5 indicating moderate quality, and scores ≤3 indicating low quality.

### 2.6 Statistical analysis

Statistical analyses were conducted via the “meta” and “metafor” packages in R statistical software (version 4.2.1) (Viechtbauer, 2010). For the meta-analysis, a generic inverse variance pooling method was employed, and the pooled effect sizes were calculated via a random effects model, using the DerSimonian-Laird approach (DerSimonian and Laird, 2015). Given that the outcome measures in this study often involve various units of measurement, in line with previous research suggestions, this study prioritized the use of standardized mean differences (SMD) as the effect size indicator (Nagashima et al., 2019). Additionally, forest plots summarizing each outcome category were generated via the “orchard” package, providing a visual representation of the pooled estimates and heterogeneity across the studies (Nakagawa et al., 2021).

Considering the small sample sizes in most of the included studies, Hedges’ g, which is based on a precise formula, was used as the effect size, and corrections for bias were applied. The Hedges’ g values were categorized as trivial (0.2), small (0.2–0.5), medium (0.5–0.8), or large (>0.8) (Nak et al., 2017). A variety of metrics, including Cochrane’s Q, the I^2^ statistic, tau^2, and Tau, were employed to evaluate heterogeneity (Nak et al., 2017). We computed a 95% confidence interval (CI). The prediction interval (PI) was also calculated to more comprehensively reflect the potential variability in future similar studies, and multiple indicators were reported simultaneously (Nagashima et al., 2019). Recent studies and standard textbooks have predominantly advocated the I^2^ statistic as the principal indicator of heterogeneity. Consequently, our primary analysis utilized I^2^, interpreting its values as follows: 0%–25% potentially insignificant; 25%–50% indicative of moderate heterogeneity; 50%–75% suggestive of substantial heterogeneity; and 75%–100% reflective of considerable heterogeneity (Cumpston et al., 2019). To evaluate the statistical power of the pooled effect estimates and minimize type II error, power analyses were conducted via the “metameta” package (Quintana, 2023).

Furthermore, subgroup analyses and meta-regressions were conducted to examine potential moderators and explore sources of heterogeneity across both categorical and continuous variables. These analyses focused on three primary domains: participant characteristics, intervention characteristics, and training protocol parameters. Based on the theoretical rationale and data availability, four key subgroup variables were selected, including age group (≤12 years vs. >12 years), weight status (normal weight [BMI <25 kg m^-2^], overweight/obese [BMI ≥25 kg m^-2^], or N/A), intervention week (<12 weeks vs. ≥12 weeks), and training frequency (≤2 sessions/week vs. >2 sessions/week). To ensure interpretability and sufficient statistical power, subgroup analyses were performed only when each subgroup contained at least five studies (Ruppar, 2020). Furthermore, subgroup analysis was considered appropriate only when moderate-to-high heterogeneity (I^2^ > 25%) was observed regarding the overall pooled effect estimates.

Finally, publication bias was assessed via contour-enhanced funnel plots and Egger’s regression test, with a p value >0.05 considered indicative of no significant publication bias (Egger et al., 1997). Sensitivity analysis was performed using a one-study-removed (leave-one-out) method, whereby each study was sequentially excluded to evaluate its influence on the overall pooled estimate.

### 2.7 Certainty of evidence

The evidence of effectiveness for each study was combined with the quality scores for use in discussing the results. The Grading of Recommendations Assessment, Development, and Evaluation (GRADE) methodology was used to rate the certainty of the evidence as “high”, “moderate”, “low”, or “very low” (Schünemann et al., 2019). GRADE was completed by two researchers, with differences resolved through consensus. This comprehensive assessment rates evidence as follows: (1) the risk of bias, downgraded by one level if “some concerns” and two levels if “high risk” of bias; (2) inconsistency, downgraded by one level when the impact of statistical heterogeneity (I^2^) is moderate (>25%) and by two levels when high >75%; (3) imprecision, downgraded by one level when statistical power <80% and if there was no clear direction of the effects (Guyatt et al., 2011); and (4) risk of publication bias, downgrade one level if Egger’s test <0.05.

## 3 Results

### 3.1 Studies retrieved

**FIGURE 1:**
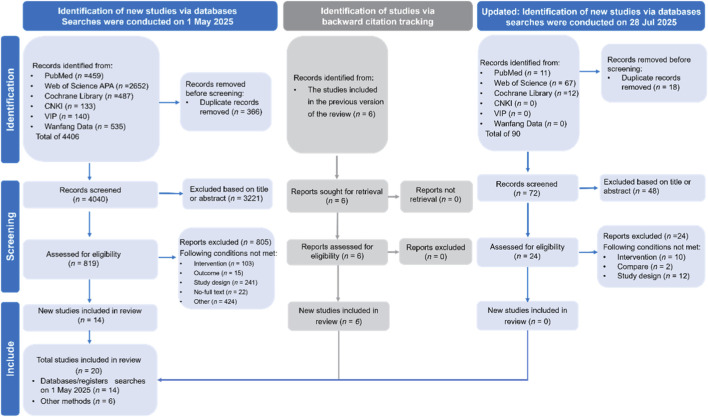
PRISMA flow diagram of study selection.

A literature search was conducted in six databases: PubMed, Web of Science APA, the Cochrane Library, and three Chinese databases: CNKI (China National Knowledge Infrastructure), VIP (Chinese Scientific Journals Database), and Wanfang Data. The initial search, conducted on 1 May 2025, identified 4406 records. After removing 366 duplicates, 4040 records remained for the title and abstract screening. An additional 6 studies were identified through backward citation tracking of a previous version of the review. The full selection process is shown in Figure 1.

### 3.2 Characteristics of included studies

Of the 20 included studies (Zheng et al., 2025; Skoradal et al., 2018; Orntoft et al., 2016; Morgado et al., 2023; Mohammed et al., 2023; Mohammed et al., 2021; Cvetkovic et al., 2018; Vasconcellos et al., 2016; Vasconcellos et al., 2021; Trajkovic et al., 2020; Seabra et al., 2016a; Seabra et al., 2016b; Seabra et al., 2014; Ryom et al., 2022; Larsen et al., 2023; Larsen et al., 2018; Krustrup et al., 2014; Hansen et al., 2013; Hammami et al., 2017; Faude et al., 2010), 16 were randomized controlled trials (RCTs). A total of 2,906 participants across all studies were aged 8–17 years. Of the participants, 1,524 (52.44%) were male, 1,174 (40.40%) were female, and 208 (7.16%) did not clearly report their gender. Participants’ BMIs ranged widely, typically between 18.6 and 29.5, with some studies focusing on overweight and obese individuals. In terms of baseline health, participants included individuals of healthy weight, overweight, and obese, with some studies specifically including individuals with metabolic health issues such as type 2 diabetes or hypertension. Overall, the study population reflected a diverse range of health conditions and weight status. Across all studies, 2,054 participants received a recreational football intervention, while 852 participants were assigned to a control condition. The duration of the intervention programs ranged from 6 to 32 weeks, with interventions delivered 2–4 times per week. Most included studies provided detailed information on participant characteristics, study design, and intervention protocol, with a few studies providing specific details of the intervention. Among the specific details provided, seven studies reported exercise intensity, which was primarily between 75% and 80% of HR_max_; 11 studies reported net session duration (excluding warm-up and cool-down), which was primarily between 30 and 90 min. No adverse events were reported across the studies. A summary of the baseline characteristics of the participants in each study is presented in Table 1.

**TABLE 1:** Characteristics of the included studies.

Author	Group	Type	N	Males (%)	Age	Height	Weight	BMI	Comorbidity	Intervention	Fre	Intensity	Sets	Duration per Set (min)	Intermittent recovery (min)	Total (min)	Number of competitors
Zheng et al. (2025)	SSG	RCT	14	100	15.1	165.4	77.4	28.3	n/a	6 weeks	2	n/a	4	4	2	16	3v3
	CON		15	100	15.1	168.5	79.5	28.0		6 weeks	2						
Morgado et al. (2023)	SSG	RCT	26	46.15	8.6	130.2	31.9	18.7	n/a	12 weeks	2	n/a	n/a	n/a	n/a	n/a	n/a
	CON		25	60	8.6	133.3	32.8	18.3		12 weeks	2						
Mohammed et al. (2023)	SSG	RCT	10	100	17.8	156.0	58.2	23.7	Type 1 diabetes	12 weeks	2	n/a	2	45	n/a	90	3v3-5v5
	CON		10	100	14.4	161.0	61.1	23.5		12 weeks	2						
Larsen et al. (2023)	SSG	RCT	61	59.01	10.7	148.1	41.3	n/a	n/a	11 weeks	2	n/a	n/a	n/a	n/a	n/a	n/a
	CON		47	55.32	10.7	149.5	40.9	n/a		11 weeks	2						
Ryom et al. (2022)	SSG	RCT	944	53	11.6	151.4	43.0	18.7	n/a	11 weeks	2	n/a	n/a	n/a	n/a	n/a	n/a
	CON		178	50	11.4	150.7	43.5	19.0		11 weeks	2						
Vasconcellos et al. (2021)	SSG	RCT	6	33.33	13.9	160.9	85.1	30.5	Chronic	12 weeks	3	n/a	1	40	n/a	40	2v2-4v4
	CON		7	28.57	14.7	163.1	84.7	30.8		12 weeks	3						
Mohammed et al. (2021)	SSG	RCT	10	100	17.8	156.0	58.2	23.7	Type 1 diabetes	12 weeks	2	n/a	2	45	n/a	90	3v3-5v5
	CON		10	100	14.4	161.0	61.1	23.5		12 weeks	2						
Trajkovic et al. (2020)	SSG	RCT	54	74.01	15.7	176.5	66.4	21.0	n/a	32 weeks	2	n/a	n/a	n/a	n/a	30	n/a
	CON		51	68.63	15.8	174.0	65.1	20.7		32 weeks	2						
Skoradal et al. (2018)	SSG	RCT	292	50	11.1	150.6	44.2	19.3	n/a	11 weeks	2	72%HRmax	n/a	n/a	n/a	n/a	3v3、4v4
	CON		100	57	11.1	149.0	42.4	18.9		11 weeks	2						
Cvetkovic et al. (2018)	SSG	RCT	10	100	12.0	157.9	63.7	25.4	n/a	12 weeks	3	n/a	4	8	2	32	5v5-7v7
	CON		14	100	12.0	162.7	67.4	25.3		12 weeks	3						
Larsen et al. (2018)	SSG	RCT	93	n/a	9.3	138.4	32.8	n/a	n/a	10 months	3	75%HRmax	n/a	n/a	n/a	n/a	3v3、4v4
	CON		115	n/a	9.3	138.2	32.7	n/a		10 months	3						
Hammami et al. (2017)	SSG	RCT	11	100	15.8	171.0	56.0	n/a	n/a	8 weeks	2	n/a	1	n/a	n/a	45	4 × 4–7 × 7
	CON		11	100	16.0	170.0	54.4	n/a		8 weeks	2						
Vasconcellos et al. (2016)	SSG	RCT	10	80	14.1	163.1	82.2	30.3	n/a	12 weeks	3	85%HRmax	n/a	n/a	n/a	40	2v2-4v4
	CON		10	60	14.8	161.2	86.3	32.2		12 weeks	3						
Seabra et al. (2016a)	SSG	CT	9	100	10.7	150.0	52.5	22.9	n/a	6 months	4	n/a	n/a	n/a	n/a	60	n/a
	CON		8	100	9.5	141.0	50.6	25.3		6 months	4						
Seabra et al. (2016b)	SSG	CT	29	100	10.5	147.5	52.5	23.7	n/a	6 months	3	78%HRmax	n/a	n/a	n/a	60	n/a
	CON		30	100	10.0	145.3	53.6	25.1		6 months	3						
Orntoft et al. (2016)	SSG	RCT	386	51.81	11.1	150.1	41.2	18.2	n/a	11 weeks	2	n/a	n/a	n/a	n/a	n/a	3v3
	CON		140	40.71	11.0	151.4	41.7	18.1		11 weeks	2						
Seabra et al. (2014)	SSG	CT	12	100	10.3	147.2	50.3	22.9	n/a	5 months	4	80%HRmax	n/a	n/a	n/a	n/a	n/a
	CON		8	100	10.6	147.1	60.7	27.7		5 months	4						
Krustrup et al. (2014)	SSG	RCT	46	50	9.4	137.0	32.0	16.9	n/a	10 weeks	3	n/a	n/a	n/a	n/a	n/a	3v3
	CON		51	41.18	9.3	138.0	32.5	16.9		10 weeks	3						
Hansen et al. (2013)	SSG	CT	20	85	10.0	146.0	50.1	23.1	n/a	3 months	4	80%HRmax	n/a	n/a	n/a	n/a	n/a
	CON		11	63.64	10.0	146.0	60.9	28.0		3 months	4						
Faude et al. (2010)	SSG	RCT	11	54.55	10.8	155.7	65.7	26.9	n/a	6 months	3	78% HRmax	n/a	n/a	n/a	30	n/a
	CON		11	72.73	10.8	157.1	64.5	26.0		6 months	3						

N, sample size; Age, mean age of participants (years); BMI, body mass index (kg/m^2^); SSG, recreational Football; CON, control; N/A, not reported; Fre, training frequency (sessions per week); HRmax, maximal heart rate.

### 3.3 Meta-analysis results

#### 3.3.1 Body composition

**FIGURE 2:**
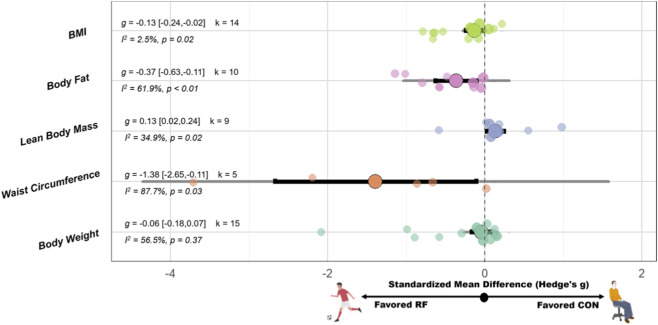
Orchard plot of the effects of recreational football on body composition. Note: Abbreviation: BMI, body mass index; g: Hedges’s g, the effect size indicator used in the pooled analysis; I^2^, quantitative indicator of heterogeneity; k, number of studies included in the pooled effect size; p-value, statistical significance level for pooled results; 95% CI, 95% confidence interval; Negative values indicate favorable effects of recreational football compared with control conditions.

SSG have significant improvement on BMI(SMD = −0.13 [−0.24, −0.02], p = 0.02, I^2^ = 2.5%, p = 0.42, Egger’s p = 0.09), lean body mass (SMD = 0.13 [0.02, 0.24], p = 0.02, I^2^ = 34.9%, p = 0.14), body fat (SMD = −0.37 [−0.63, −0.11], p < 0.01, I^2^ = 61.9%, p < 0.01, Egger’s p < 0.01), and waist circumference (WC) (SMD = −1.38 [−2.65, −0.11], p = 0.03, I^2^ = 87.7%, p < 0.01) compared with the CON. However, no significant effect on body weight (SMD = −0.06 [−0.18, 0.07], p = 0.37; I^2^ = 56.5%, p < 0.01, Egger’s p = 0.04) compared with the CON. A schematic diagram of the orchards summarizing each outcome indicator is shown in Figure 2.

**FIGURE 3:**
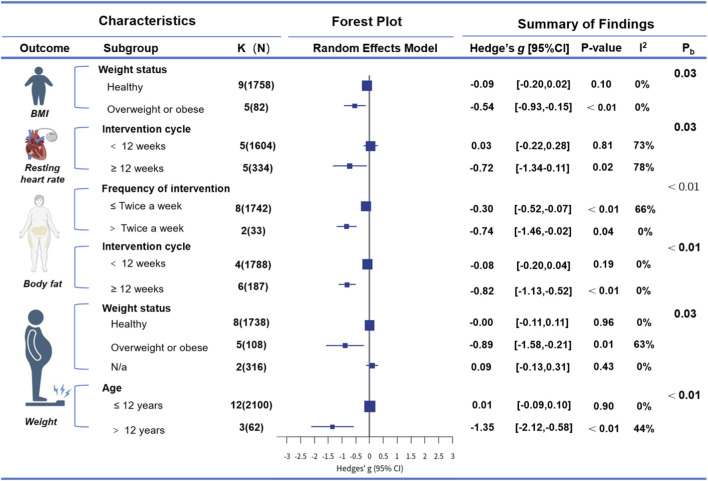
Summary of subgroup analysis. Note: Hedge’s g, the effect size indicators used in the pooled; I^2^, quantitative indicators of heterogeneity; K, the total number of effects included in the pooled effect size; N, sample size of each study; P-value: statistically significant p values for pooled results; P_d_, indicates the p-value for subgroup differences, testing whether the effect sizes differ significantly between subgroups; The area of the square represents the weight of each study, the horizontal line represents the 95% confidence interval, and the vertical line represents the null effect (effect size = 0). Negative values indicate that the recreational football intervention has a beneficial effect compared with the control group.

Subgroup analyses indicated that weight status significantly moderated the effect on BMI (Pb < 0.03). Participants who were overweight or obese exhibited greater reductions in BMI (g = −0.54 [–0.93, −0.15]), whereas no significant effect was observed among those without notable weight gain (g = −0.09 [–0.20, 0.02]). For body fat, both intervention duration (Pb < 0.01) and frequency (Pb < 0.01) emerged as significant moderators. Interventions lasting longer than 12 weeks produced more pronounced reductions (g = −0.82 [–1.13, −0.52]) compared with shorter interventions (≤12 weeks: g = −0.08 [–0.20 to 0.04]). Regarding body weight, weight status (Pb = 0.03) and age (Pb < 0.01) significantly influenced outcomes. A greater decrease in body weight was observed in participants who were overweight or obese (g = −0.89 [–1.58, −0.21]), whereas no significant change was found in healthy individuals (g = 0.00 [–0.11, 0.11]). No significant moderating effects were identified for the remaining variables. Please see Figure 3 for detailed subgroup analysis information.

#### 3.3.2 Cardiometabolic health

**FIGURE 4:**
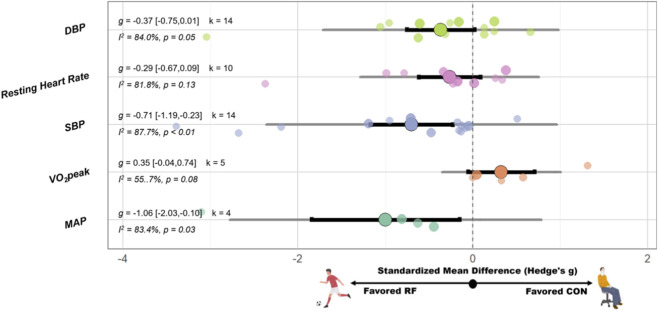
Orchard plot of the effects of recreational football on Blood pressure and cardiopulmonary function-related indicators. Note: Abbreviation: DBP, diastolic blood pressure; SBP, systolic blood pressure; MAP, mean arterial pressure; g, Hedges’s g, the effect size indicator used in the pooled analysis; I^2^, quantitative indicator of heterogeneity; k, number of studies included in the pooled effect size; p-value, statistical significance level for pooled results; 95% CI: 95% confidence interval; Negative values indicate favorable effects of recreational football compared with control conditions (except for VO_2_peak).

**FIGURE 5:**
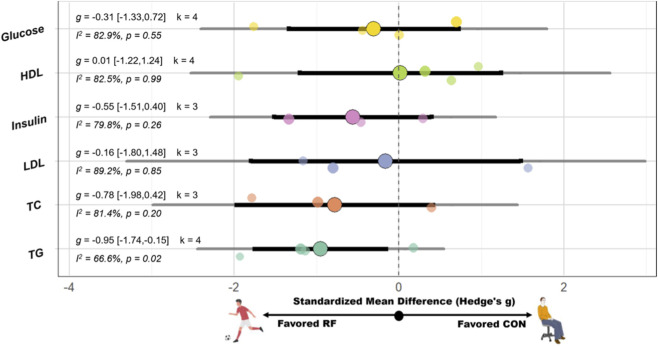
Orchard plot of the effects of recreational football on Metabolic and blood lipid-related. Note: Abbreviation: HDL, high-density lipoprotein; LDL, low-density lipoprotein; TC, total cholesterol; TG, triglycerides; g: Hedges’s g, the effect size indicator used in the pooled analysis; I^2^, quantitative indicator of heterogeneity; k: number of studies included in the pooled effect size; p-value, statistical significance level for pooled results; 95% CI, 95% confidence interval; Negative values indicate favorable effects of recreational football compared with control conditions.

SSG have significant improvement on mean arterial pressure (MAP) (SMD = −1.06 [−2.03, −0.10], p = 0.03, I^2^ = 83.4%, p < 0.01), SBP (SMD = −0.71 [−1.19, −0.23], p < 0.01, I^2^ = 87.7%, p < 0.01, Egger’s p = 0.54), and TG (SMD = −0.95 [−1.74, −0.15], p = 0.02, I^2^ = 66.6%, p = 0.03) compared with the CON. However, no significant effect on DBP (SMD = −0.37 [−0.75, 0.01], p = 0.05; I^2^ = 84.0%, p < 0.01, Egger’s p = 0.62), resting heart rate (SMD = −0.29 [−0.67, 0.09], p = 0.13; I^2^ = 81.8%, p < 0.01, Egger’s p = 0.16), VO_2_peak (SMD = 0.35 [−0.04, 0.74], p = 0.08; I^2^ = 55.7%, p = 0.06), high-density lipoprotein (SMD = 0.01 [−1.22, 1.24], p = 0.99; I^2^ = 82.5%, p < 0.01), LDL (SMD = −0.16 [−1.80, 1.48], p = 0.85; I^2^ = 89.2%, p < 0.01), glucose (SMD = −0.31 [−1.33, 0.72], p = 0.55; I^2^ = 82.9%, p < 0.01), insulin (SMD = −0.55 [−1.51, 0.40], p = 0.26; I^2^ = 79.8%, p < 0.01), and total cholesterol (TC) (SMD = −0.78 [−1.98, 0.42], p = 0.20; I^2^ = 81.4%, p < 0.01) compared with the CON. A schematic diagram of the orchards summarizing each outcome indicator is shown in Figures 4, 5.

Subgroup analysis revealed that intervention duration significantly moderated the effect on resting heart rate (Pb = 0.03). Interventions lasting ≥12 weeks were associated with greater reductions in BMI (g = −0.72 [–1.34, −0.11]), whereas no significant effect was observed for interventions shorter than 12 weeks (g = 0.03 [–0.22, 0.28]). No significant moderating effects were identified for the other variables. Please see Figure 3 for detailed subgroup analysis information.

#### 3.3.3 Functional performance

**FIGURE 6:**
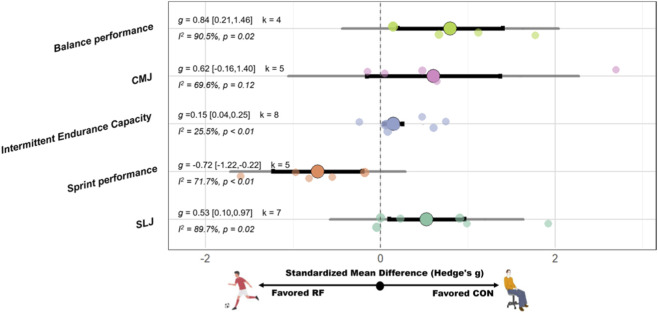
Orchard plot of the effects of recreational football on functional performance. Note: Abbreviation: CMJ, countermovement jump; SLJ, standing long jump; g: Hedges’s g, the effect size indicator used in the pooled analysis; I^2^, quantitative indicator of heterogeneity; k, number of studies included in the pooled effect size; p-value, statistical significance level for pooled results; 95% CI, 95% confidence interval; Positive values indicate a beneficial effect of recreational football compared to the control condition (except for sprint performance).

**FIGURE 7:**
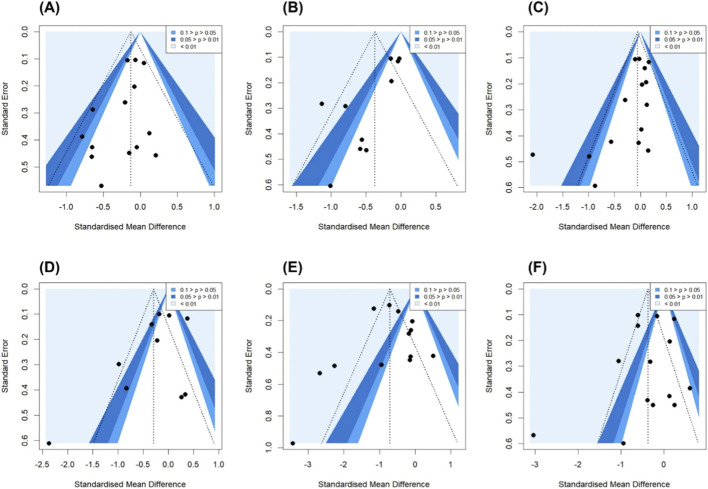
Funnel plot. Note: Abbreviation: SBP, Systolic blood pressure; DBP, diastolic blood pressure. **(A)** BMI, **(B)** Body fat. **(C)** Body weight. **(D)** Resting heart rate. **(E)** SBP. **(F)** DBP.

SSG have significant improvement on intermittent endurance capacity (SMD = 0.15 [0.04, 0.25], p < 0.01, I^2^ = 25.5%, p = 0.23), sprint performance (SMD = −0.72 [−1.22, −0.22], p < 0.01, I^2^ = 71.7%, p < 0.01), standing long jump (SLJ) (SMD = 0.53 [0.10, 0.97], p = 0.02, I^2^ = 89.7%, p < 0.01), and balance performance (SMD = 0.84 [0.21, 1.46], p = 0.02, I^2^ = 90.5%, p < 0.01) compared with the CON. However, no significant effect on CMJ (SMD = 0.62 [−0.16, 1.40], p = 0.12; I^2^ = 69.6%, p = 0.01) compared with the CON. See Figure 7 for funnel plots of risk of publication bias for each outcome. A schematic diagram of the orchards summarizing each outcome indicator is shown in Figure 6. For detailed forest plots, please refer to Figure 6.

### 3.4 Sensitivity analysis

Sensitivity analyses indicated that the results of several outcomes were influenced by individual studies. For body weight, the overall effect was not significant (SMD ≈ −0.20), but heterogeneity markedly decreased from 82%–86%–4% after excluding Zheng 2025. For CMJ, the overall effect was non-significant (SMD = 0.62, p = 0.12); however, it became significant after excluding Seabra 2016 (SMD = 0.37, p = 0.01, I^2^ = 0%). Similarly, the overall effect of insulin was not significant (SMD = −0.55, p = 0.26), but reached significance following the removal of Mohammed 2021 (SMD = −0.97, p = 0.03, I^2^ = 62%). For TC, the pooled effect was not significant (SMD = −0.78, p = 0.20), yet became significantly reduced after excluding Mohammed 2021 (SMD = −1.25, p < 0.01, I^2^ = 41%). Finally, VO_2_peak showed an overall positive trend (SMD = 0.35, p = 0.08), which became significant after excluding Ryom 2022 (SMD = 0.52, p < 0.01, I^2^ = 28%).

### 3.5 Risk of bias and methodological quality

**FIGURE 8:**
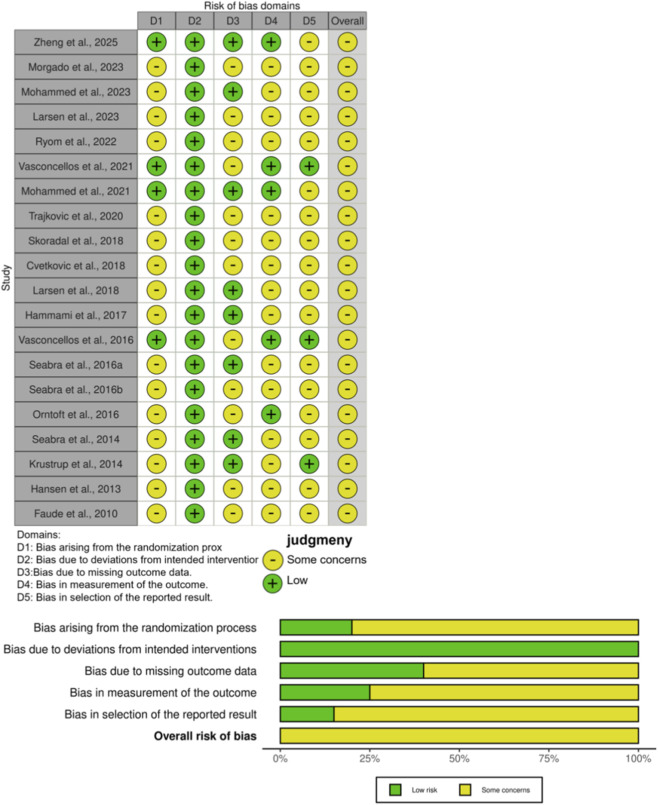
Risk-of-bias assessment diagram.

The risk of bias for each study was reported (Figure 8). Overall, all studies (100%) indicated “some concerns”. 80% of studies did not report allocation concealment and were therefore considered to have “some risk” in the randomization process. 60% of studies had some degree of participant dropout and were therefore considered to have “some concerns”. Most studies (80%) did not report strict blinding of outcome assessment and were therefore considered to have “some concerns”.

The methodological quality of the included studies was evaluated via the Physiotherapy Evidence Database (PEDro) scale, as shown in Table 2. The average PEDro score of all studies was 5.4, and the overall quality of the included literature was high, with 35% being of high quality (≥6 points) and 65% being of moderate quality (4-5 points).

**TABLE 2:** PEDro scale assessment results.

Author et al., year	D1	D2	D3	D4	D5	D6	D7	D8	D9	D10	D11	Total
Zheng et al. (2025)	Y	1	1	1	0	0	1	1	1	1	1	8
Morgado et al. (2023)	Y	1	0	0	0	0	0	1	1	1	1	5
Mohammed et al. (2023)	Y	1	0	0	0	0	0	1	1	1	1	5
Larsen et al. (2023)	N	1	0	0	0	0	0	1	1	1	1	5
Ryom et al. (2022)	Y	1	0	0	0	0	0	0	1	1	1	4
Vasconcellos et al. (2021)	Y	1	1	1	0	0	1	0	1	1	1	7
Mohammed et al. (2021)	Y	1	1	1	0	0	1	1	1	1	1	8
Trajkovic et al. (2020)	N	1	0	0	0	0	0	1	1	1	1	5
Skoradal et al. (2018)	Y	1	0	0	0	0	0	1	1	1	1	5
Cvetkovic et al. (2018)	Y	1	0	1	0	0	0	0	1	0	1	4
Larsen et al. (2018)	Y	1	0	0	0	0	0	1	1	1	1	5
Hammami et al. (2017)	Y	1	0	1	0	0	0	1	1	1	1	6
Vasconcellos et al. (2016)	Y	1	1	1	0	0	1	0	1	1	1	7
Seabra et al. (2016a)	Y	0	0	1	0	0	0	1	1	1	1	5
Seabra et al. (2016b)	Y	0	0	1	0	0	0	1	1	1	1	5
Orntoft et al. (2016)	N	1	0	0	0	0	1	1	1	1	1	6
Seabra et al. (2014)	Y	1	0	1	0	0	0	1	1	1	1	6
Morgado et al. (2023)	Y	0	0	0	0	0	0	1	1	1	1	4
Krustrup et al. (2014)	Y	1	0	0	0	0	0	1	1	1	1	5
Hansen et al. (2013)	Y	0	0	0	0	0	0	1	1	1	1	4
Faude et al. (2010)	Y	1	0	0	0	0	0	0	1	1	1	4

Studies scoring ≥6 is considered high quality, those scoring 4–5 are considered moderate quality, and those scoring ≤3 are considered low quality.

1. Eligibility criteria were specified (not included in the total score).

2. Subjects were randomly allocated to groups (in a crossover study et al., subjects were randomly allocated an order in which treatments were received).

3. Allocation was concealed.

4. The groups were similar at baseline regarding the most important prognostic indicators.

5. There was blinding of all subjects.

6. There was blinding of all therapists who administered the therapy.

7. There was blinding of all assessors who measured at least one key outcome.

8. Measures of at least one key outcome were obtained from more than 85% of the subjects initially allocated to groups.

9. All subjects for whom outcome measures were available received the treatment or control condition as allocated or et al., where this was not the case et al., data for at least one key outcome was analysed by “intention to treat”.

10. The results of between-group statistical comparisons are reported for at least one key outcome.

11. The study provides both point measures and measures of variability for at least one key outcome.

### 3.6 Results of the certainty of the evidence

Overall, the certainty of the evidence ranged from very low to low across the assessed outcomes, as shown in Table 3. Low certainty evidence was identified for BMI, intermittent endurance capacity, and sprint performance. In contrast, most other outcomes including body weight, body fat percentage, fat free mass, WC, blood pressure (SBP, DBP, and mean arterial), resting heart rate, lipid profiles (TC, LDL, HDL, TG), glucose metabolism (fasting glucose and insulin), VO_2_peak, and most functional performance measures (CMJ, SLJ, balance) were supported by very low certainty evidence. These downgrades mainly reflected risks of bias, inconsistency due to high heterogeneity, and imprecision.

**TABLE 3:** GRADE.

Outcome	No of participants (studies)	Certainty assessment					Standardized mean effect (95% CI)†	GRADE^a^
		Risk of bias	Inconsistency	Indirectness	Imprecision	Other		
Body composition								
Body Weight	15 (12 RCTs)	Serious	Serious	Serious	Serious	None	**−0.06 (-0.18 to 0.07)**	⨁◯◯◯ VERY LOW
BMI	14 (11 RCTs)	Serious	Not serious	Serious	Not serious	None	**−0.13 (-0.24 to -0.02)**	⨁⨁◯◯ LOW
Body Fat Percentage	10 (8 RCTs)	Serious	Serious	Not serious	Serious	None	**−0.37 (-0.63 to -0.11)**	⨁◯◯◯ VERY LOW
Fat Free Mass	9 (7 RCTs)	Serious	Serious	Serious	Not serious	None	**0.13 (0.02 to 0.24)**	⨁◯◯◯ VERY LOW
WC	5 (4 RCTs)	Serious	Very serious	Serious	Not serious	Large Effect Size	**−1.38 (-2.65 to -0.11)**	⨁◯◯◯ VERY LOW
Cardiometabolic health								
SBP	14 (12 RCTs)	Serious	Very serious	Not serious	Not serious	None	**−0.71 (-1.19 to -0.23)**	⨁◯◯◯ VERY LOW
DBP	14 (12 RCTs)	Serious	Very serious	Serious	Not serious	None	**−0.37 (-0.75 to 0.01)**	⨁◯◯◯ VERY LOW
MAP	4 (4 RCTs)	Serious	Very serious	Serious	Not serious	Large effect size	**−1.06 (-2.03 to -0.10)**	⨁◯◯◯ VERY LOW
Resting Heart Rate	10 (9 RCTs)	Serious	Very serious	Serious	Not serious	None	**−0.29 (-0.67 to 0.09)**	⨁◯◯◯ VERY LOW
VO2peak	5 (4 RCTs)	Serious	Serious	Serious	Not serious	None	**0.35 (-0.04 to 0.74)**	⨁◯◯◯ VERY LOW
TC	3 (2 RCTs)	Serious	Very serious	Serious	Not serious	None	**−0.78 (-1.98 to 0.42)**	⨁◯◯◯ VERY LOW
LDL	3 (2 RCTs)	Serious	Very serious	Serious	Not serious	None	**−0.16 (-1.80 to 1.48)**	⨁◯◯◯ VERY LOW
HDL	4 (3 RCTs)	Serious	Very serious	Serious	Not serious	None	**0.01 (-1.22 to 1.24)**	⨁◯◯◯ VERY LOW
TG	4 (3 RCTs)	Serious	Serious	Serious	Not serious	Large effect size	**−0.95 (-1.74 to -0.15)**	⨁◯◯◯ VERY LOW
Fasting Glucose	4 (3 RCTs)	Serious	Very serious	Serious	Not serious	None	**−0.31 (-1.33 to 0.72)**	⨁◯◯◯ VERY LOW
Fasting Insulin	3 (2 RCTs)	Serious	Very serious	Serious	Not serious	None	**−0.55 (-1.51 to 0.40)**	⨁◯◯◯ VERY LOW
Functional performance								
CMJ	5 (4 RCTs)	Serious	Serious	Serious	Not serious	None	**0.62 (-0.16 to 1.40)**	⨁◯◯◯ VERY LOW
SLJ	7 (7 RCTs)	Serious	Serious	Serious	Not serious	None	**0.53 (0.10 to 0.97)**	⨁◯◯◯ VERY LOW
Intermittent endurance capacity	8 (7 RCTs)	Serious	Not serious	Serious	Not serious	None	**0.15 (0.04 to 0.25)**	⨁⨁◯◯ LOW
Sprint performance	4 (3 RCTs)	Serious	Serious	Not serious	Not serious	None	**−0.72 (-1.22 to -0.22)**	⨁⨁◯◯ LOW
Balance performance	4 (4 RCTs)	Serious	Serious	Serious	Not serious	Large effect size	**0.84 (0.21 to 1.46)**	⨁◯◯◯ VERY LOW

^a^ Certainty of evidence according to Grading of Recommendations, Assessment, Development and Evaluations (GRADE).

High: We are very confident in the estimated effect.

Moderate: Our confidence in the estimated effect is moderate.

Low: We have limited confidence in the estimated effect.

Very low: We have very little confidence in the estimated efect

BMI, body mass index; WC: waist circumference; SBP: systolic blood pressure; DBP: diastolic blood pressure; MAP: mean arterial pressure; TC: total cholesterol; LDL: low-density lipoprotein cholesterol; HDL: high-density lipoprotein cholesterol; TG: triglycerides; CMJ: countermovement jump; SLJ: standing long jump.

Standardized mean effect : effect size (SMD) after the data have been pooled in the meta-analysis.

## 4 Discussion

This meta-analysis systematically evaluated the effects of recreational football interventions on body composition, cardiometabolic health, and functional performance in untrained children and adolescents. Overall, participants experienced favorable changes in weight management, fat reduction, and blood pressure regulation, along with improvements in physical performance indicators such as endurance, speed, and balance. These improvements align with the intermittent, multi-directional, and competitive load of recreational football: small-field competition and frequent starts and stops can simultaneously increase cardiopulmonary load and neuromuscular recruitment, and support the reduction of blood pressure and the improvement of physical fitness through the adaptation of autonomic nervous and vascular functions (Stephen et al., 2011). Although certain metabolic markers did not show significant improvement, consistent with previous pediatric exercise studies, we observed similar improvements in body composition, blood pressure, and physical fitness. Importantly, no adverse events were reported across the included studies, supporting the safety of recreational football in youth populations. The results highlight the potential of recreational football as an integrative strategy for school-based physical education and community health promotion.

### 4.1 Body composition

This study demonstrated that recreational football significantly improved body composition in adolescents, as reflected by reductions in BMI, body fat, WC, and lean mass, with no significant change in overall body weight. These findings suggest that, despite the absence of significant weight loss, participants achieved a healthier body composition, characterized by fat reduction accompanied by gains in lean mass, which may have masked changes in total body weight (Bonilla et al., 2024). Our results are consistent with the meta-analysis by Milanović et al. (2019), which included 31 controlled studies. Compared to non-exercise controls, recreational soccer showed a significant benefit in fat mass (−1.7 kg) and a slight increase in lean body mass. However, it showed inconsistent results in BMI, fat mass, and lean body mass compared to running or Zumba, which is related to the high heterogeneity of the population and the program (Milanović et al., 2019).

This effect may be attributed to the high energy expenditure and muscular stimulation generated by the intermittent, high-intensity movements inherent in football, such as sprinting, abrupt stops, and changes of direction (Dellal et al., 2010). By contrast, a systematic review by Clemente et al. (2022) in adolescents reported limited evidence for improvements in body composition, likely due to short intervention periods or the predominance of normal-weight participants, which affected the observed effects (Clemente et al., 2022). Similarly, a meta-analysis by Gómez-Álvarez et al. (2024) did not find significant reductions in BMI or body weight. However, their forest plot revealed a downward trend in WC (*p* = 0.05), which, although not statistically significant, was directionally consistent with our findings (Gómez-Álvarez et al., 2024). It is worth noting that the result became significant after conducting a sensitivity analysis, indicating that the conclusions are influenced by the number and heterogeneity of studies. Our findings complement this evidence, demonstrating for the first time a significant reduction in WC in adolescents, suggesting that recreational football exerts a powerful effect on abdominal obesity (Liu et al., 2022; Cheng et al., 2024). This reduction may be explained by the intense mobilization of abdominal fat through continuous alternating aerobic and anaerobic activity, which promotes visceral fat loss.

Clinically, even small decreases in waist circumference and BMI may reduce the future risk of obesity-related conditions (Ross et al., 2020; Sanguankeo et al., 2017). For example, for every 1 cm decrease in waist circumference, the risk of developing metabolic syndrome decreases by approximately 9% (Hosseinpanah et al., 2010); Meanwhile, for every 1 kg/m^2^ decrease in BMI, the risk of developing type 2 diabetes is associated with a decrease of approximately 15% (Chen et al., 2018). This finding is clinically relevant, as WC is closely associated with visceral fat accumulation, and reductions in visceral fat have been shown to substantially lower the risk of metabolic syndrome, type 2 diabetes, and cardiovascular disease (Ryan and Yockey, 2017; Dhar et al., 2025). Moreover, the observed increase in lean mass suggests that recreational football training not only facilitates fat loss but also stimulates favorable muscular adaptations. Repeated sprinting, jumping, and body contact activities are known to enhance type II muscle fiber hypertrophy, even in youth populations, through increased mechanical load and tension (Andersen et al., 2016; Faigenbaum et al., 2009). Repeated sprinting, jumping, and contact activities enhance muscle fiber hypertrophy and neuromuscular coordination, thereby contributing to increases in lean body mass (Xie et al., 2024). Additionally, electromyography (EMG) studies have demonstrated that such dynamic and high-intensity movements improve neuromuscular coordination by enhancing motor unit recruitment and synchronization, which may partly explain the observed improvements in lean mass and functional capacity (Mohr et al., 2015). In the long term, greater muscle mass can improve insulin sensitivity, elevate basal metabolic rate, and reduce the risk of cardiovascular and metabolic diseases (McPherron et al., 2013; Braith and Stewart, 2006).

Notably, our subgroup analysis demonstrated that overweight or obese adolescents benefited more substantially than their normal-weight peers (BMI: g = −0.54 vs. −0.09; weight: g = −0.89 vs. 0.00), indicating that baseline weight status is an important moderating factor. In contrast, the previous meta-analysis by Gómez-Álvarez et al. (2024) did not perform subgroup analyses of body composition, thereby failing to identify potential moderating factors (Gómez-Álvarez et al., 2024). This effect may be explained by the greater fat reserves and poorer metabolic profiles of obese adolescents, which, when exposed to high-intensity, interval-based football training, facilitate lipolysis and induce pronounced metabolic adaptations, leading to greater reductions in weight and body fat (Jabbour et al., 2011). These findings indicate that recreational football may serve as a promising intervention for obese adolescents, with the potential to improve cardiometabolic profiles and thereby reduce future health risks. Moreover, intervention duration and frequency emerged as additional key moderators. Programs lasting at least 12 weeks and delivered at a frequency of two or more sessions per week produced significant reductions in body fat, whereas shorter or less frequent interventions were largely ineffective. This may reflect the fact that improvements in fat metabolism and increases in muscle mass require a sustained cumulative effect before becoming stably expressed (Mika et al., 2019). For clinical and public health practice, these results highlight the importance of implementing recreational football interventions in schools and communities at a long-term and regular frequency to ensure that the benefits translate into meaningful health outcomes.

In conclusion, recreational football may contribute to improvements in some body composition outcomes among adolescents, with potentially greater benefits observed in individuals with obesity and those receiving higher-frequency interventions. While some of these trends did not reach statistical significance, they may still indicate meaningful patterns that warrant further investigation. These observations could inform the design of more targeted and dose-sensitive health promotion programs, though future studies with larger sample sizes and more standardized protocols are needed to confirm these effects.

### 4.2 Cardiometabolic health

This study found that recreational football significantly reduced MAP, SBP, and TG, but did not achieve statistical significance for DBP, resting heart rate, VO_2_peak, TC, LDL, HDL, blood glucose, or insulin.

These findings are largely consistent with previous research. Milanović et al. (2019) reported that football training lowered SBP (−4.2 mmHg), DBP (−3.9 mmHg), and resting heart rate (−6 bpm), while significantly increasing VO_2_peak (Milanović et al., 2019). Sarmento et al. (2020) proposed the concept of “football as medicine,” systematically incorporating 44 empirical studies (with no design restrictions) covering healthy and chronically ill adults, with comprehensive evidence pointing to benefits in multiple aspects such as blood pressure, blood lipids, and insulin sensitivity (Sarmento et al., 2020). In contrast, although DBP did not reach statistical significance in our study, the direction of improvement was consistent. VO_2_peak also showed no significant improvement, which may be related to the relatively short duration and moderate intensity of the included interventions. Previous trials have reported that sustained high-intensity or longer-term training is often required to induce stable improvements in adolescent cardiorespiratory fitness (Bangsbo et al., 2016). Gómez-Álvarez et al. (2024) included 16 studies involving 2,872 children and adolescents, and their results showed a significant decrease in blood pressure and triglycerides, while no significant change was observed in blood glucose, consistent with our findings (Sarmento et al., 2020). This may be attributable to the fact that adolescents are naturally active and possess relatively high baseline cardiopulmonary function, making short-term or moderate-intensity interventions insufficient to yield significant improvements in VO_2_peak. Although VO_2_peak improvements were not statistically significant, the observed positive trend suggests that extended intervention duration or higher training intensity may ultimately yield meaningful gains in cardiorespiratory fitness (Lackland et al., 2018).

By contrast, blood pressure and lipid levels appear more sensitive to exercise stimuli, and even moderate-intensity football training can induce measurable improvements (LeBlanc and Janssen, 2010). A decrease in triglyceride levels further indicates a reduced risk of atherosclerosis. A decrease in triglyceride levels further indicates a reduced risk of atherosclerosis (Zhang et al., 2022). Moreover, the absence of significant changes in HDL and blood glucose may be partly explained by the relatively healthy baseline profiles of most participants, which left limited physiological room for further improvement. In non-clinical adolescent populations, homeostatic regulation of glucose metabolism and lipid transport is typically well maintained, and moderate exercise may not produce marked alterations unless baseline dyslipidemia or insulin resistance is present. Additionally, intervention intensity and total training volume in the included studies were moderate, which may have been insufficient to trigger the metabolic adaptations necessary for meaningful increases in HDL or improvements in glucose tolerance.

Unlike the meta-analysis by Gómez-Álvarez et al. (2024), we identified intervention duration and population characteristics as potential moderating factors. Our subgroup analysis indicated that interventions lasting at least 12 weeks significantly reduced resting heart rate, whereas shorter interventions produced no significant effect. This finding suggests that adaptations of the cardiorespiratory system require time to accumulate, and that regular medium-to long-term training can induce stable adjustments in autonomic regulation and cardiac function. Moreover, obese adolescents exhibited greater improvements in resting heart rate than their healthy weight peers, consistent with the findings of Li et al. (2025). This may be attributable to their higher baseline sympathetic activity and reduced parasympathetic tone (Abel et al., 2019). Moreover, obese adolescents exhibited greater improvements in resting heart rate compared with their healthy-weight peers, likely due to their higher baseline sympathetic activity and lower parasympathetic tone (Abel et al., 2019). Consequently, they may experience larger increases in parasympathetic activity and reductions in sympathetic activity following exercise intervention, leading to greater decreases in resting heart rate. Clinically, reductions in resting heart rate are highly relevant to adolescent health. Epidemiological evidence indicates that a decrease of 3-4 beats per minute is associated with a lower risk of future cardiovascular events and all-cause mortality (Zhang et al., 2016). Thus, even in adolescents without overt metabolic abnormalities, regular participation in recreational football may contribute to long-term cardiovascular protection through reductions in resting heart rate.

In summary, this study further validates the role of recreational football in improving blood pressure and blood lipids in adolescents, while suggesting limited effects on VO_2_peak and glucose metabolism. These results highlight the importance of intervention duration and population characteristics: achieving more stable cardiorespiratory adaptation requires at least medium-to long-term intervention, and obese adolescents may be among the groups most benefiting from football intervention.

### 4.3 Functional performance

This study is the first to systematically quantify the effects of recreational football on functional performance in adolescents. We observed significant improvements in interval endurance, sprint speed, SLJ, and balance, whereas vertical jump did not reach statistical significance. These findings extend previous evidence on the functional benefits of recreational football. Clemente et al. (2022) included 17 randomized controlled trials, covering aerobic, strength, speed, change of direction, and health-related outcomes. However, due to high heterogeneity in outcomes and protocols, they mainly used qualitative synthesis and did not conduct quantitative analysis on indicators such as speed and balance (Clemente et al., 2022). Gómez-Álvarez et al. (2024) similarly did not detect significant improvements in muscle strength in their primary analysis; however, sensitivity analyses excluding studies with small effect sizes or high heterogeneity revealed a statistically significant effect on muscle strength, although measures of speed and agility were not included (Gómez-Álvarez et al., 2024). Milanović et al. (2019) also reported improvements in CMJ (+2.27 cm) following football training, although their analysis did not include balance or endurance measures (Milanović et al., 2019). By presenting quantitative estimates across diverse performance outcomes, the present study not only complements prior evidence but also fills an important gap by demonstrating the multidimensional effects of recreational football on adolescent functional performance.

The observed benefits may be attributed to long-term neuromuscular stimulation through football training. Repeated rapid directional changes and contact movements enhance motor unit recruitment and coordination, translating into improvements in speed, lower-limb explosive power, and balance (Asadi et al., 2017). These adaptations are likely mediated by specific neuromotor mechanisms, including increased corticospinal excitability, enhanced proprioceptive feedback, and improved intermuscular synchronization. Furthermore, repeated deceleration-acceleration cycles and frequent unanticipated directional shifts stimulate sensorimotor pathways involved in postural control and reactive agility (Sport-Specific Balance, 2025). These changes are similar to the core elements of neuromuscular training (NMT/INT) or sensorimotor training for adolescents, helping to more effectively activate type II muscle fibers and improve the rate of strength development, which is particularly important for dynamic balance and sprint performance. Meta-analyses have shown that body-weight NMT programs alone can significantly improve dynamic balance, coordination, and motor control in adolescent athletes (SMD ≈0.79, 95% CI [0.38, 1.20]) (Yin et al., 2023). Improvements in interval endurance likely result from repeated high-intensity running interspersed with moderate-intensity recovery (Yin et al., 2024b), which progressively enhances both cardiorespiratory and muscular endurance. These physiological gains are supported by increased mitochondrial density, improved capillarization, and upregulation of oxidative enzyme activity, contributing to greater aerobic efficiency and fatigue resistance (Krustrup et al., 2010).

By contrast, the absence of significant improvement in vertical jump may reflect insufficient training stimuli specifically targeting vertical explosive power or high heterogeneity across studies. These findings hold important clinical and practical implications. Gains in speed and balance reflect enhanced neuromuscular coordination, which may reduce the risk of sports injuries and improve adolescents’ capacity to engage in daily activities and physical exercise. More specifically, improvements in agility and balance suggest enhanced anticipatory postural adjustments and more rapid transitions between eccentric and concentric phases of movement, which are essential for injury prevention and functional performance (Emery et al., 2015). Improvements in SLJ performance indicate increased lower-limb explosive strength, directly associated with overall physical fitness and athletic performance. Enhancements in endurance measures suggest that recreational football not only improves strength and agility but also promotes synergistic gains in cardiorespiratory and metabolic fitness.

In summary, interventions lasting at least 12 weeks resulted in more stable improvements in functional performance, suggesting the need for long-term, regular training. Training design variables (such as field size (e.g., area per player), number of players, and work-rest ratio) should be adjusted according to the target outcome; different configurations may have different effects on specific performance indicators, which warrants further investigation. The results of this study demonstrate that recreational football can improve functional performance in adolescents across multiple dimensions, addressing the lack of quantitative evidence in previous reviews and providing empirical support for school-based physical education and youth health promotion.

### 4.4 Limitations and future directions

Several limitations should be acknowledged when interpreting the findings of this meta-analysis. First, the included studies exhibited substantial heterogeneity, likely attributable to variations in participant characteristics (e.g., age, sex, and weight status) and differences in intervention modalities and study designs. Second, although most trials were of moderate methodological quality, several lacked proper blinding, allocation concealment, or intention-to-treat analyses, thereby introducing potential risk of bias. Third, the relatively small sample sizes across studies may have reduced the statistical power and increased the risk of type II errors. Fourth, publication bias cannot be entirely excluded, as studies with non-significant or unfavorable results may have been underrepresented in the literature, potentially inflating the pooled estimates. Fifth, the fidelity and consistency of intervention implementation were inconsistently reported, with many studies failing to provide detailed descriptions of training parameters (e.g., field size, number of players, intensity, and session frequency), which precluded subgroup or meta-regression analyses to examine dose–response relationships. Finally, the GRADE assessment indicated that most outcomes were supported by low or very low certainty of evidence, suggesting that current conclusions should be interpreted with caution until more rigorously designed and transparently reported trials become available.

Future research should aim to address these limitations. First, larger and more rigorously designed randomized controlled trials are required to strengthen the reliability of conclusions. Second, standardization and transparent reporting of football intervention protocols (including field size, number of players, training intensity, and training content) should be encouraged to reduce inter-study heterogeneity and facilitate subgroup and regression analyses. Third, intervention periods should be extended, and follow-up assessments conducted to determine whether short-term improvements translate into long-term health benefits, while also evaluating adherence and safety. In particular, future trials should include systematic and standardized reporting of adverse events and injury surveillance to ensure comprehensive assessments of intervention safety. This will improve transparency and allow for more accurate risk-benefit evaluations in youth populations. Fourth, future trials should incorporate predefined subgroup analyses stratified by sex, age, baseline fitness, and weight status to better understand individual variability in training response. Such analyses may help identify which populations benefit most from recreational football, and uncover sex- or age-specific neuromuscular or metabolic adaptations that can guide precision-based exercise prescription. Fifth, more comprehensive and multidimensional outcome measures spanning not only functional performance and body composition but also neuromotor coordination, agility-specific adaptations, and metabolic biomarkers should be employed to elucidate underlying mechanisms and enhance explanatory power. Finally, there is an urgent need to develop and validate football-based exercise programs that can be widely implemented in school settings. Such programs should not only effectively improve the physical health of children and adolescents but also be feasible, engaging, and integrable into school curricula, thereby promoting sustainable health benefits with substantial implications for both educational practice and public health policy (Yin et al., 2024a; Yin et al., 2024c; Yin et al., 2025).

### 4.5 Practical implications

The results of this study indicate that recreational football is an intervention with significant health-promoting potential. It may be incorporated into school sports and community health programs where feasible and context-appropriate, without imposing additional financial burden, thereby providing a practical approach to weight management and fitness improvement for adolescents, particularly those who were overweight or obese. With respect to exercise prescription, a recent meta-analysis of recreational football has suggested that optimal interventions might last at least 12 weeks, with 2-3 sessions per week of approximately 45–60 min each (Li et al., 2025). Such programs have typically included the FIFA 11+ warm-up (∼10 min) (Bizzini and Dvorak, 2015), small-sided games (3v3-5v5; 4–6 min per bout with 2-min recovery intervals; total duration 20–30 min; intensity gradually increasing to 80% of maximum heart rate), and a 5–10 min cool-down with stretching. Evidence from heterogeneous trials suggests that this structured format may reduce body fat and blood pressure and enhance functional performance, although the overall certainty of evidence is low to very low (GRADE). It may also improve adherence through its enjoyable and social nature, thereby potentially amplifying public health benefits. Recreational football thus has the potential to serve as a scalable strategy for preventing obesity, reducing cardiometabolic risk, and improving physical health in adolescents. Moreover, although the study included non-school-organized leisure activities, considering the effectiveness and safety of the intervention measures, we believe that the core value of this study is to provide evidence-based support for policymakers and education departments to incorporate leisure football into school sports and health promotion programs, and promote the widespread development of leisure football in schools and communities, rather than being limited to parks or spontaneous leisure activities. Finally, this recommendation aligns with our subgroup analysis, which demonstrated greater benefits among obese adolescents, further underscoring its value for key populations.

## 5 Conclusions

Evidence of low to moderate certainty suggests that recreational football is an effective and feasible exercise intervention for improving body composition, reducing cardiometabolic risk factors such as blood pressure and lipid levels, and enhancing functional performance, including speed, endurance, and balance among children and adolescents. Subgroup analyses indicate that these effects are more pronounced in who were overweight or obese, with more consistent improvements in body fat and cardiorespiratory fitness observed when interventions last at least 12 weeks and are performed more than twice per week. While these benefits are supported by small to moderate effect sizes, the overall certainty of evidence is low to moderate due to methodological limitations, heterogeneous intervention protocols, and small sample sizes. Nonetheless, given its engaging, social, and adaptable nature, recreational football demonstrates strong potential for promoting participation and adherence among youth within school and community health programs. Future research should prioritize large-scale, high-quality randomized controlled trials and evaluate strategies for the systematic and safe integration of recreational football into school curricula to strengthen the evidence base and optimize its role in adolescent health promotion.

## Data Availability

The original contributions presented in the study are included in the article/Supplementary Material, further inquiries can be directed to the corresponding author.

## References

[B1] Abel P. F. Migueles J. H. Jose M. G. Pablo M. G. Maria R. A. Cristina C. S. (2019). Frontiers | heart rate is a better predictor of cardiorespiratory fitness than heart rate variability in overweight/obese children: the ActiveBrains project. Exerc. Physiol. 10.3389/fphys.2019.00510 PMC6514130 31133870

[B2] Ahmad T. H. M. Kusuma IDMAW Pramono B. A. Ramadhani M. I. Nirwansyah W. T. (2025). Unveiling anaerobic soccer training: comparing its effects with small-sided games on youth performance enhancement. Pedagogy Phys. Cult. Sports 29, 150–159. 10.15561/26649837.2025.0301

[B3] Andersen T. R. Schmidt J. F. Pedersen M. T. Krustrup P. Bangsbo J. (2016). The effects of 52 weeks of soccer or resistance training on body composition and muscle function in +65-Year-Old healthy males – a randomized controlled trial. PLOS ONE 11, e0148236. 10.1371/journal.pone.0148236 26886262 PMC4757560

[B4] Asadi A. Arazi H. Ramirez-Campillo R. Moran J. Izquierdo M. (2017). Influence of maturation stage on agility performance gains after plyometric training: a systematic review and meta-analysis. J. Strength and Cond. Res. 31, 2609–2617. 10.1519/JSC.0000000000001994 28557853

[B5] Bangsbo J. Krustrup P. Duda J. Hillman C. Andersen L. B. Weiss M. (2016). The copenhagen consensus conference 2016: children, youth, and physical activity in schools and during leisure time. Br. J. Sports Med. 50, 1177–1178. 10.1136/bjsports-2016-096325 27354718 PMC5036221

[B6] Bizzini M. Dvorak J. (2015). FIFA 11+: an effective programme to prevent football injuries in various player groups worldwide—a narrative review. Br. J. Sports Med. 49, 577–579. 10.1136/bjsports-2015-094765 25878073 PMC4413741

[B7] Bonilla D. A. Petro J. L. Cannataro R. Kreider R. B. Stout J. R. (2024). Editorial: new insights and advances in body recomposition. Front. Nutr. 11, 1467406. 10.3389/fnut.2024.1467406 39290563 PMC11405322

[B8] Braith R. W. Stewart K. J. (2006). Resistance exercise training. Circulation 113, 2642–2650. 10.1161/circulationaha.105.584060 16754812

[B9] Chen Y. Zhang X. P. Yuan J. Cai B. Wang X. L. Wu X. L. (2018). Association of body mass index and age with incident diabetes in Chinese adults: a population-based cohort study. BMJ Open 8, e021768. 10.1136/bmjopen-2018-021768 30269064 PMC6169758

[B10] Cheng B. Du J. Tian S. Zhang Z. Chen W. Liu Y. (2024). High-intensity interval training or lactate administration combined with aerobic training enhances visceral fat loss while promoting VMH neuroplasticity in female rats. Lipids Health Dis. 23, 405. 10.1186/s12944-024-02397-2 39696579 PMC11653782

[B11] Clemente F. M. Moran J. Ramirez-Campillo R. Oliveira R. Brito J. Silva A. F. (2022). Recreational soccer training effects on pediatric populations physical fitness and health: a systematic review. Child. (Basel) 9, 1776. 10.3390/children9111776 36421225 PMC9689246

[B12] Cumpston M. Li T. Page M. J. Chandler J. Welch V. A. Higgins J. P. (2019). Updated guidance for trusted systematic reviews: a new edition of the cochrane handbook for systematic reviews of interventions. Cochrane Database Syst. Rev. 2019, ED000142. 10.1002/14651858.ED000142 31643080 PMC10284251

[B13] Cvetkovic N. Stojanovic E. Stojiljkovic N. Nikolic D. Scanlan A. T. Milanovic Z. (2018). Exercise training in overweight and obese children: recreational football and high-intensity interval training provide similar benefits to physical fitness. Scand. J. Med. Sci. Sports 28, 18–32. 10.1111/sms.13241 29979479

[B14] Dellal A. Keller D. Carling C. Chaouachi A. Wong D. P. Chamari K. (2010). Physiologic effects of directional changes in intermittent exercise in soccer players. J. Strength and Cond. Res. 24, 3219–3226. 10.1519/JSC.0b013e3181b94a63 19996785

[B15] DerSimonian R. Laird N. (2015). Meta-analysis in clinical trials revisited. Contemp. Clin. Trials 45, 139–145. 10.1016/j.cct.2015.09.002 26343745 PMC4639420

[B16] Dhar D. Packer J. Michalopoulou S. Cruz J. Stansfield C. Viner R. M. (2025). Assessing the evidence for health benefits of low-level weight loss: a systematic review. Int. J. Obes. 49, 254–268. 10.1038/s41366-024-01664-7 39487296 PMC11805710

[B17] Egger M. Smith G. D. Schneider M. Minder C. (1997). Bias in meta-analysis detected by a simple, graphical test. BMJ 315, 629–634. 10.1136/bmj.315.7109.629 9310563 PMC2127453

[B18] Emery C. A. Roy T. O. Whittaker J. L. Nettel-Aguirre A. van Mechelen W. (2015). Neuromuscular training injury prevention strategies in youth sport: a systematic review and meta-analysis. Br. J. Sports Med. 49, 865–870. 10.1136/bjsports-2015-094639 26084526

[B19] Faigenbaum A. D. Kraemer W. J. Blimkie C. J. R. Jeffreys I. Micheli L. J. Nitka M. (2009). Youth resistance training: updated position statement paper from the national strength and conditioning association. J. Strength Cond. Res. 23, S60–S79. 10.1519/JSC.0b013e31819df407 19620931

[B20] Faude O. Kerper O. Multhaupt M. Winter C. Beziel K. Junge A. (2010). Football to tackle overweight in children. Scand. J. Med. Sci. Sports 20, 103–110. 10.1111/j.1600-0838.2009.01087.x 20136766

[B21] Garber C. E. Blissmer B. Deschenes M. R. Franklin B. A. Lamonte M. J. American College of Sports Medicine (2011). American college of sports medicine position stand. Quantity and quality of exercise for developing and maintaining cardiorespiratory, musculoskeletal, and neuromotor fitness in apparently healthy adults: guidance for prescribing exercise. Med. Sci. Sports Exerc 43, 1334–1359. 10.1249/MSS.0b013e318213fefb 21694556

[B22] Gómez-Álvarez N. Boppre G. Hermosilla-Palma F. Reyes-Amigo T. Oliveira J. Fonseca H. (2024). Effects of small-sided soccer games on physical fitness and cardiometabolic health biomarkers in untrained children and adolescents: a systematic review and meta-analysis. J. Clin. Med. 13, 5221. 10.3390/jcm13175221 39274434 PMC11396522

[B23] Guthold R. Stevens G. A. Riley L. M. Bull F. C. (2020). Global trends in insufficient physical activity among adolescents: a pooled analysis of 298 population-based surveys with 1·6 million participants. Lancet Child and Adolesc. Health 4, 23–35. 10.1016/S2352-4642(19)30323-2 31761562 PMC6919336

[B24] Guyatt G. H. Oxman A. D. Kunz R. Brozek J. Alonso-Coello P. Rind D. (2011). Rating the quality of evidence—imprecision. J. Clin. Epidemiol. 64, 1283–1293. 10.1016/j.jclinepi.2011.01.012 21839614

[B25] Hammami A. Kasmi S. Razgallah M. Tabka Z. Shephard R. J. Bouhlel E. (2017). Recreational soccer training improves heart-rate variability indices and physical performance in untrained healthy adolescent. Sport Sci. Health 13, 507–514. 10.1007/s11332-016-0343-4

[B26] Hansen P. R. Andersen L. J. Rebelo A. N. Brito J. Hornstrup T. Schmidt J. F. (2013). Cardiovascular effects of 3 months of football training in overweight children examined by comprehensive echocardiography: a pilot study. J. Sports Sci. 31, 1432–1440. 10.1080/02640414.2013.792951 23829576

[B27] Hosseinpanah F. Barzin M. Mirmiran P. Azizi F. (2010). Effect of changes in waist circumference on metabolic syndrome over a 6.6-year follow-up in Tehran. Eur. J. Clin. Nutr. 64, 879–886. 10.1038/ejcn.2010.79 20485305

[B28] Jabbour G. Lemoine-Morel S. Casazza G. A. Hala Y. Moussa E. Zouhal H. (2011). Catecholamine response to exercise in obese, overweight, and lean adolescent boys. Med. Sci. Sports Exerc 43, 408–415. 10.1249/MSS.0b013e3181f1bef3 20689461

[B29] Kallio P. Pahkala K. Heinonen O. J. Tammelin T. H. Pälve K. Hirvensalo M. (2021). Physical inactivity from youth to adulthood and adult cardiometabolic risk profile. Prev. Med. 145, 106433. 10.1016/j.ypmed.2021.106433 33497685

[B30] Krustrup P. Aagaard P. Nybo L. Petersen J. Mohr M. Bangsbo J. (2010). Recreational football as a health promoting activity: a topical review. Scand. J. Med. Sci. Sports 20 (Suppl. 1), 1–13. 10.1111/j.1600-0838.2010.01108.x 20210908

[B31] Krustrup P. Hansen P. R. Nielsen C. M. Larsen M. N. Randers M. B. Manniche V. (2014). Structural and functional cardiac adaptations to a 10‐week school‐based football intervention for 9–10‐year‐old children. Scand. Med. Sci. Sports 24, 4–9. 10.1111/sms.12277 24944128

[B32] Krustrup P. Williams C. A. Mohr M. Hansen P. R. Helge E. W. Elbe A. M. (2018). The “Football is Medicine” platform—scientific evidence, large-scale implementation of evidence-based concepts and future perspectives. Scand. J. Med. and Sci. Sports 28, 3–7. 10.1111/sms.13220 29917263

[B33] Lackland D. T. Carey R. M. Conforto A. B. Rosendorff C. Whelton P. K. Gorelick P. B. (2018). Implications of recent clinical trials and hypertension guidelines on stroke and future cerebrovascular research. Stroke 49, 772–779. 10.1161/STROKEAHA.117.019379 29467237 PMC5829017

[B34] Larsen M. N. Nielsen C. M. Madsen M. Manniche V. Hansen L. Bangsbo J. (2018). Cardiovascular adaptations after 10 months of intense school‐based physical training for 8‐ to 10‐year‐old children. Scand. Med. Sci. Sports 28, 33–41. 10.1111/sms.13253 30047176

[B35] Larsen M. N. Terracciano A. Møller T. K. Aggestrup C. S. Buono P. Krustrup P. (2023). An 11-week school-based “health education through football” programme improves musculoskeletal variables in 10–12-yr-old Danish school children. Bone Rep. 18, 101681. 10.1016/j.bonr.2023.101681 37187574 PMC10176027

[B36] LeBlanc A. G. Janssen I. (2010). Dose-response relationship between physical activity and dyslipidemia in youth. Can. J. Cardiol. 26, e201–e205. 10.1016/s0828-282x(10)70400-1 20548982 PMC2903992

[B37] Li S. Li H. Wang B. Zeng Z. Zhang R. Yan H. (2025). Effects of recreational football on body composition and cardiometabolic health in overweight or Obese individuals: a systematic review and meta-analysis. Life (Basel) 15, 1276. 10.3390/life15081276 40868925 PMC12387140

[B38] Liu Y. Li Y. Cheng B. Feng S. Zhu X. Chen W. (2022). Comparison of visceral fat lipolysis adaptation to high-intensity interval training in obesity-prone and obesity-resistant rats. Diabetology and Metabolic Syndrome 14, 62. 10.1186/s13098-022-00834-9 35501906 PMC9063201

[B39] McPherron A. C. Guo T. Bond N. D. Gavrilova O. (2013). Increasing muscle mass to improve metabolism. Adipocyte 2, 92–98. 10.4161/adip.22500 23805405 PMC3661116

[B40] Mika A. Macaluso F. Barone R. Di Felice V. Sledzinski T. (2019). Effect of exercise on fatty acid metabolism and adipokine secretion in adipose tissue. Front. Physiol. 10, 26. 10.3389/fphys.2019.00026 30745881 PMC6360148

[B41] Milanović Z. Pantelić S. Čović N. Sporiš G. Mohr M. Krustrup P. (2019). Broad-spectrum physical fitness benefits of recreational football: a systematic review and meta-analysis. Br. J. Sports Med. 53, 926–939. 10.1136/bjsports-2017-097885 29371223 PMC6662951

[B42] Mohammed M. H. H. Al-Qahtani M. H. H. Takken T. (2021). Effects of 12 weeks of recreational football (soccer) with caloric control on glycemia and cardiovascular health of adolescent boys with type 1 diabetes. Pediatr. Diabetes 22, 625–637. 10.1111/pedi.13203 33745203

[B43] Mohammed M. H. H. Al-Qahtani M. H. H. Takken T. (2023). Health-related fitness of adolescent boys with type 1 diabetes mellitus after recreational football exercise with caloric control. Rev. Diabetic Studies 19, 77–85. 10.1900/RDS.2023.19.77

[B44] Mohr M. Nann M. von Tscharner V. Eskofier B. Nigg B. M. (2015). Task-dependent intermuscular motor unit synchronization between medial and lateral vastii muscles during dynamic and isometric squats. PLoS One 10, e0142048. 10.1371/journal.pone.0142048 26529604 PMC4631473

[B45] Morgado M. C. Sousa M. Coelho A. B. Vale S. Costa J. A. Seabra A. (2023). Effects of ‘Football and Nutrition for Health’ program on body composition, physical fitness, eating behaviours, nutritional knowledge, and psychological status among 7 to 10 years school children. Front. Pediatr. 11, 15. 10.3389/fped.2023.1251053 38027281 PMC10663241

[B46] Nagashima K. Noma H. Furukawa T. A. (2019). Prediction intervals for random-effects meta-analysis: a confidence distribution approach. Stat. Methods Med. Res. 28, 1689–1702. 10.1177/0962280218773520 29745296

[B47] Nakagawa S. Noble D. W. A. Senior A. M. Lagisz M. (2017). Meta-evaluation of meta-analysis: ten appraisal questions for biologists. BMC Biol. 15, 18. 10.1186/s12915-017-0357-7 28257642 PMC5336618

[B48] Nakagawa S. Lagisz M. O’Dea R. E. Rutkowska J. Yang Y. Noble D. W. A. (2021). The orchard plot: cultivating a forest plot for use in ecology, evolution, and beyond. Res. Synth. Methods 12, 4–12. 10.1002/jrsm.1424 32445243

[B49] Orntoft C. Fuller C. W. Larsen M. N. Bangsbo J. Dvorak J. Krustrup P. (2016). FIFA 11 for health’ for Europe. II: effect on health markers and physical fitness in Danish schoolchildren aged 10-12 years. Br. J. Sports Med. 50, 1394–1399. 10.1136/bjsports-2016-096124 27130927 PMC5136709

[B50] Page M. J. McKenzie J. E. Bossuyt P. M. Boutron I. Hoffmann T. C. Mulrow C. D. (2021). The PRISMA 2020 statement: an updated guideline for reporting systematic reviews. BMJ 372, n71. 10.1136/bmj.n71 33782057 PMC8005924

[B51] Quintana D. S. (2023). A guide for calculating study-level statistical power for meta-analyses. Adv. Methods Pract. Psychol. Sci. 6, 25152459221147260. 10.1177/25152459221147260

[B52] Ross R. Neeland I. J. Yamashita S. Shai I. Seidell J. Magni P. (2020). Waist circumference as a vital sign in clinical practice: a consensus statement from the IAS and ICCR working group on visceral obesity. Nat. Rev. Endocrinol. 16, 177–189. 10.1038/s41574-019-0310-7 32020062 PMC7027970

[B53] Ruppar T. (2020). Meta-analysis: how to quantify and explain heterogeneity? Eur. J. Cardiovasc. Nurs. 19, 646–652. 10.1177/1474515120944014 32757621

[B54] Ryan D. H. Yockey S. R. (2017). Weight loss and improvement in comorbidity: differences at 5%, 10%, 15%, and over. Curr. Obes. Rep. 6, 187–194. 10.1007/s13679-017-0262-y 28455679 PMC5497590

[B55] Ryom K. Christiansen S. R. Elbe A. M. Aggestrup C. S. Madsen E. E. Madsen M. (2022). The Danish ‘11 for Health’ program raises health knowledge, well-being, and fitness in ethnic minority 10-to 12-year-olds. Scand. J. Med. Sci. Sports 32, 138–151. 10.1111/sms.14057 34555200

[B56] Sanguankeo A. Lazo M. Upala S. Brancati F. L. Bonekamp S. Pownall H. J. (2017). Effects of visceral adipose tissue reduction on CVD risk factors independent of weight loss: the look AHEAD study. Endocr. Res. 42, 86–95. 10.1080/07435800.2016.1194856 27351077 PMC5573136

[B57] Sarmento H. Clemente F. M. Marques A. Milanovic Z. Harper L. D. Figueiredo A. (2020). Recreational football is medicine against non‐communicable diseases: a systematic review. Scand. J. Med. Sci. Sports 30, 618–637. 10.1111/sms.13611 31834941

[B58] Schünemann H. J. Higgins J. P. Vist G. E. Glasziou P. Akl E. A. Skoetz N. (2019). “On behalf of the cochrane GRADEing methods group (formerly applicability and recommendations methods group) and the cochrane statistical methods group. 2019. Completing ‘Summary of findings’ tables and grading the certainty of the evidence,” in Cochrane handbook for systematic reviews of interventions. Chichester, England: John Wiley and Sons, Ltd, 375–402.

[B59] Sciences U of SDF of H (2025). Football proven to prevent and treat lifestyle diseases. News-Medical.

[B60] Seabra A. C. Seabra A. F. Brito J. Krustrup P. Hansen P. R. Mota J. (2014). Effects of a 5‐month football program on perceived psychological status and body composition of overweight boys. Scand. Med. Sci. Sports 24, 10–16. 10.1111/sms.12268 24944129

[B61] Seabra A. Serra H. Seabra A. Brito J. Krustrup P. Mota J. (2016a). Effects of A 6-Month football intervention program on bone mass and physical fitness in overweight children. Spine Res. 02. 10.21767/2471-8173.100010

[B62] Seabra A. Katzmarzyk P. Carvalho M. J. Seabra A. Coelho-E-Silva M. Abreu S. (2016b). Effects of 6-month soccer and traditional physical activity programmes on body composition, cardiometabolic risk factors, inflammatory, oxidative stress markers and cardiorespiratory fitness in obese boys. J. Sports Sci. 34, 1822–1829. 10.1080/02640414.2016.1140219 26890580

[B63] Skoradal M. B. Purkhús E. Steinholm H. Olsen M. H. Orntoft C. Larsen M. N. (2018). FIFA 11 for health’ for Europe in the Faroe Islands: effects on health markers and physical fitness in 10-to 12-year-old schoolchildren. Scand. J. Med. Sci. Sports 28, 8–17. 10.1111/sms.13209 29882318

[B64] Sport-Specific Balance (2025). Sports Med. 10.1007/s40279-013-0130-1 24293269

[B65] Stephen H. H. Brian D. Franco I. Aaron C. (2011). Physiology of small-sided games training in football: a systematic review. Sports Medicine Auckl. NZ 41, 199–220. 10.2165/11539740-000000000-00000 21395363

[B66] Swift D. L. Johannsen N. M. Lavie C. J. Earnest C. P. Church T. S. (2014). The role of exercise and physical activity in weight loss and maintenance. Prog. Cardiovasc Dis. 56, 441–447. 10.1016/j.pcad.2013.09.012 24438736 PMC3925973

[B67] The Global Status Report (2022). On physical activity.

[B68] Trajkovic N. Madic D. M. Milanovic Z. Macak D. Padulo J. Krustrup P. (2020). Eight months of school-based soccer improves physical fitness and reduces aggression in high-school children. Biol. Sport 37, 185–193. 10.5114/biolsport.2020.94240 32508386 PMC7249792

[B69] Vasconcellos F. Seabra A. Cunha F. Montenegro R. Penha J. Bouskela E. (2016). Health markers in Obese adolescents improved by a 12-week recreational soccer program: a randomised controlled trial. J. Sports Sci. 34, 564–575. 10.1080/02640414.2015.1064150 26208409

[B70] Vasconcellos F. Cunha F. A. Gonet D. T. Farinatti P. T. V. (2021). Does recreational soccer change metabolic syndrome status in Obese adolescents? A pilot study. Res. Q. Exerc Sport 92, 91–99. 10.1080/02701367.2019.1711007 32083979

[B71] Viechtbauer W. (2010). Conducting meta-analyses in R with the metafor package. J. Stat. Softw. 36, 1–48. 10.18637/jss.v036.i03

[B72] Xie L. Chen J. Dai J. Zhang W. Chen L. Sun J. (2024). Exploring the potent enhancement effects of plyometric training on vertical jumping and sprinting ability in sports individuals. Front. Physiol. 15, 1435011. 10.3389/fphys.2024.1435011 39318363 PMC11420559

[B73] Yin M. Chen Z. Nassis G. P. Liu H. Li H. Deng J. (2023). Chronic high-intensity interval training and moderate-intensity continuous training are both effective in increasing maximum fat oxidation during exercise in overweight and obese adults: a meta-analysis. J. Exerc. Sci. and Fit. 21, 354–365. 10.1016/j.jesf.2023.08.001 37701124 PMC10494468

[B79] Yin M. Deng S. Chen Z. Zhang B. Zheng H. Bai M. (2024a). Exercise snacks are a time-efficient alternative to moderate-intensity continuous training for improving cardiorespiratory fitness but not maximal fat oxidation in inactive adults: a randomized controlled trial. Appl. Physiol. Nutr. Metab. 49 (7), 920–932. 10.1139/apnm-2023-0593 38569204

[B74] Yin M. Li H. Bai M. Liu H. Chen Z. Deng J. (2024b). Is low-volume high-intensity interval training a time-efficient strategy to improve cardiometabolic health and body composition? A meta-analysis. Appl. Physiol. Nutr. Metab. 49, 273–292. 10.1139/apnm-2023-0329 37939367

[B80] Yin M. Deng S. Chen Z. Zhang B. Zheng H. Bai M. (2024c). Effects of integrating stair climbing-based exercise snacks into the campus on feasibility, perceived efficacy, and participation perspectives in inactive young adults: a randomized mixed-methods pilot study. Scand. J. Med. Sci. Sports 34 (12), e14771. 10.1111/sms.14771 39587826

[B81] YinM. LiY. AzizA. R. BuffeyA. BishopD. J. BaoD. (2025). Short bouts of accumulated exercise: Review and consensus statement on definition, efficacy, feasibility, practical applications, and future directions. J Sport Health Sci. 101088. 10.1016/j.jshs.2025.101088 40972791

[B75] Zhang D. Shen X. Qi X. (2016). Resting heart rate and all-cause and cardiovascular mortality in the general population: a meta-analysis. CMAJ 188, E53–E63. 10.1503/cmaj.150535 26598376 PMC4754196

[B76] Zhang B. H. Yin F. Qiao Y. N. Guo S. D. (2022). Triglyceride and triglyceride-rich lipoproteins in atherosclerosis. Front. Mol. Biosci. 9, 909151. 10.3389/fmolb.2022.909151 35693558 PMC9174947

[B77] Zheng B. Xu Q. Zhang J. (2025). Combining HIIT with small-sided soccer games enhances cardiometabolic and physical fitness more than each alone in overweight youth: a randomized controlled study. J. Sport Sci. Med. 24, 104–115. 10.52082/jssm.2025.104 40046214 PMC11877289

[B78] Zouhal H. Hammami A. Tijani J. M. Jayavel A. de Sousa M. Krustrup P. (2020). Effects of small-sided soccer games on physical fitness, physiological responses, and health indices in untrained individuals and clinical populations: a systematic review. Sports Med. 50, 987–1007. 10.1007/s40279-019-01256-w 31989457

